# Silicon and gadolinium co-doped hydroxyapatite/PLGA scaffolds with osteoinductive and MRI dual functions

**DOI:** 10.3389/fbioe.2023.1310017

**Published:** 2024-01-09

**Authors:** Shaodong Xie, Min Guo, Deming Zeng, Hanwen Luo, Ping Zhong, Zixuan Deng, Yu Wang, Zhiqiang Xu, Peibiao Zhang

**Affiliations:** ^1^ Department of Rehabilitation Medicine, Foshan Hospital of Traditional Chinese Medicine, Foshan, China; ^2^ Key Laboratory of Polymer Ecomaterials, Changchun Institute of Applied Chemistry, Chinese Academy of Sciences, Changchun, China; ^3^ Graduate Student of the Eighth Clinical Medical College of Guangzhou University of Chinese Medicine, Guangzhou, China

**Keywords:** osteoinductive, magnetic resonance imaging, silicon, gadolinium, co-doping, bone repair

## Abstract

**Introduction:** An ideal bone repair scaffold should have dual functions of osteoinductive ability and in *vivo* imaging. In this study, the simultaneous substitution of silicon (Si) and gadolinium (Gd) in hydroxyapatite (HA) as potential multifunctional bone graft materials has been successfully developed.

**Methods:** A series of HA nanoparticles (HA NPs) doped with different proportions of Si and Gd were prepared. The chemical structure and phase composition of the materials were analyzed using Fourier transform infrared (FTIR) spectroscopy and X-ray diffraction (XRD). The microstructure, magnetic properties, surface potential, and cytotoxicity of the materials were also analyzed. The magnetic resonance imaging (MRI) effect of Gd&Si-HA/poly(lactic-co-glycolic acid) (Gd&Si-HA/PLGA) composite materials was evaluated. Osteogenic-related gene expression, alkaline phosphatase (ALP) level, and mineralization capacity of MC3T3-E1 cultured on Gd&Si-HA/PLGA composite materials were also detected.

**Results and Discussion:** The 1.5Gd&Si-HA@PLGA group showed good ability to promote osteogenic differentiation of cells. The MRI effect of the 1.5Gd&Si-HA@PLGA scaffold was observable. This HA material containing Si and Gd co-doping has a broad application prospect in the field of bone tissue engineering owing to its ability to enhance osteoinductive property and improve MRI effect.

## 1 Introduction

In recent years, degradable polyester materials and calcium phosphate-based bioceramic composites have been developed and applied as bone repair materials (He et al., 2023; Li et al., 2023; Qian et al., 2023). Calcium phosphate-based bioceramic materials mainly include hydroxyapatite (HA) and beta-tricalcium phosphate (β-TCP). However, after implantation, the degradation rate of implants cannot be accurately and effectively monitored by computed tomography (CT) and digital radiography, and the osteogenic activity of implant materials is still far from the demand of clinical requirements.

HA is a type of calcium phosphate-based bioceramic material that is chemically similar to the inorganic components of bone and teeth. HA has high bioactivity and biocompatibility and can be combined with degradable polyester materials (such as polylactic acid (PLA), poly(lactic-co-glycolic acid) (PLGA), and polycaprolactone (PCL)) to synthesize nanocomposites, which are a promising implant material for bone repair (Sadeghi et al., 2022; Wei et al., 2022; Xu et al., 2022). However, the bone repair composite containing HA has only bone conductivity but no bone induction ability. Such implants cannot be detected by magnetic resonance imaging (MRI).

The lanthanum (Ln) series of rare earth elements have ionic radii similar to Ca^2+^, and these elements exhibit remarkable optical, electrical, magnetic, and biological activities. Among them, gadolinium (Gd) is the most widely used Ln rare earth element in clinical practice. Its 4f layer has the highest number of unpaired electrons in parallel orbits, and the spin relaxation time of Gd^3+^ can match the Larmor frequency of protons under a suitable magnetic field. Therefore, the Gd chelate (gadolinium-diethylenetriamine pentaacetic acid (Gd-DTPA)) is currently used as an MRI contrast agent in clinics (Chen et al., 2023). However, the instability of Gd chelates *in vivo* easily causes toxicity, so the search for a new type of safe and non-toxic MRI contrast agent has become a research hotspot in recent years. In addition, Gd chelates have strong water solubility and cannot be stably combined with hydrophobic degradable polyester materials. The resulting complex is limited in developing effects due to the hydrophobicity of the matrix. Therefore, it is urgent to develop safe, non-toxic MRI contrast agent materials containing Gd particles to meet the requirements of MRI and biosafety. Huang et al. (2016) combined gadolinium phosphate monohydrate (GdPO_4_.H_2_O) with HA and PLGA to obtain a novel composite material that could be imaged by MRI, but its osteogenic induction ability was insufficient.

The molecular structure of HA can accept many ion substitutions, which contributes to its new properties. Many studies have investigated the substitution of exogenous ions for HA to improve its physicochemical, functional, and biological properties *in vitro* or *in vivo* (Gene et al., 2018a; Gene et al., 2018b; Geng et al., 2021a; Geng et al., 2021b). Ln rare earth elements can replace Ca^2+^ ions in HA molecules, thus contributing to their HA photoelectromagnetic and other physical properties and biological activities. In particular, the introduction of Gd^3+^ ions as dopants is expected to enable the MRI developing properties of the composites (Chen et al., 2011).

The concentration of silicon (Si) in human plasma is 2–10 μM. Carlis and Milne reported that a lack of Si in chickens and rats will lead to bone deformation and cartilage tissue defects, which suggests that Si plays an important role in bone and cartilage metabolism and can promote the matrix synthesis of bone and cartilage (Carlisle, 1972; Schwarz and Milne, 1972). Many studies have been conducted on the influence of trace elements on bone, and it has been found that Si is related to bone calcification. The formation of acid phosphatase and alkaline phosphatase in bone metabolism and bone formation in mice with sufficient Si is higher than that in mice with insufficient Si. A dietary supplement of Si can increase the absorption of Ca in the femur (Seaborn and Nielsen, 1994). As expected, Si-HA-based materials exhibit enhanced bone apposition, bone in-growth, and cell-mediated degradation compared to stoichiometric HA controls. The phase composition of the materials is highly dependent on the Ca/(P + Si) and Ca/P ratio of the system, the level of Si addition, and the method of introducing Si to the calcium apatite (Pietak et al., 2007). Based on the changes in lattice parameters with Si inclusion and considerations of atomic radii, the simplest model of incorporation is that Si-HA accepts substitutions of SiO_4_^4-^ for PO_4_^3-^ groups. To avoid a large cost in energy, local charge neutrality is mandated, and this requires some other defects to be associated with PO_4_^3-^ to compensate for the charge deficit and caused doping dose limitation (Patel et al., 2002). Therefore, reducing charge compensation to increase Si doping dose may be an effective strategy for improving the biological activity of HA-based materials.

In this study, Gd&Si-HA nanoparticles (NPs) were prepared using the anion–cation co-doping method (Gd^3+^ replaces Ca^2+^; SiO_4_^4−^ replaces PO_3_^3−^) to address the shortcomings of calcium phosphate-based bioceramics for bone repair. PLGA and HA with different Gd and Si contents were prepared to yield composite bone repair materials, and their MRI ability was detected. Cell viability was used to assess the biocompatibility of each group of materials. The osteogenic differentiation of different groups of materials was evaluated by analyzing the expression level of osteogenic-related genes, immunofluorescence, alkaline phosphatase (ALP) activity, and calcium deposition.

## 2 Materials and methods

### 2.1 Materials

Ca(NO_3_)_2_, (NH_4_)_2_HPO_3_, Gd(NO_3_)_3_, TEOS, and Si(OC_4_H_9_)_4_ were purchased from Sinopharm Chemical Reagent Co., Ltd. PLGA (GA:LA = 75:25; molecular weight = 8 Da) were purchased from Zhongkekangdi Technology Co., Ltd.

### 2.2 Preparation of Gd&Si-HA NPs using the hydrothermal method

HA doped with different proportions of Si and Gd was synthesized using the hydrothermal method. Gd(NO_3_)_3·_6H_2_O, Ca(NO_3_)_2·_4H_2_O, (NH_4_)_2_HPO_4_, and Si(OCH_2_CH_3_) (TEOS) were used as sources of Gd, Ca, P, and Si, respectively. The amounts of reagents used in the hydrothermal reaction are shown in Supplementary Table S1. The reaction mixture was stirred in a water bath at 60°C, and pH was maintained at 10 by adding 25% ammonium solution. The reaction lasted for 1 h. Next, the reaction mixture was transferred into polytetrafluoroethylene vessels and sealed in a stainless steel autoclave, followed by hydrothermal treatment at 180°C for 12 h. After the hydrothermal reaction, the reaction systems were cooled to room temperature, and the Gd&Si-HA NPs doped with different proportions of Si and Gd were obtained. The Gd&Si-HA NPs were then separated by centrifugation (10,000 rpm, 10 min), washed several times with ethanol and deionized water, and subsequently dried at 120°C for 24 h.

### 2.3 Material characterization

Scanning electron microscopy (SEM) (ZEISS Gemini 2, Germany), X-ray diffraction (XRD) (Bruker D8 ADVANCE, Germany), and Fourier-transform infrared (FTIR) spectroscopy (Bruker INVENIO-R, Germany) were used to characterize the morphology and chemical composition of the prepared samples. The zeta potential of the prepared samples was measured by dynamic light scattering (DLS) (Zetasizer Nano ZS90, Malvern Instruments, United Kingdom). The magnetic properties of the prepared samples were characterized using a vibrating sample magnetometer (VSM; Quantum Design-MPMS-XL7).

### 2.4 Protein adsorption capacity

Different concentrations of lysozyme (LYS) and bovine serum albumin (BSA) were used to detect the protein adsorption capacity of different groups of materials. Here, 10 mg of the materials was added to 0.5, 1.0, 1.5, and 2.0 mg/mL LYS and BSA–phosphate-buffered solution (PBS), respectively. A 10-mL centrifuge tube containing nanoparticles and proteins was then incubated at 150 rpm at 37°C for 12 h. After protein adsorption, the sample was centrifuged for 10 min at 4°C at 13,000 rpm, and the protein content in the supernatant was determined using the BCA protein quantitative kit (Solarbio, China). The total protein minus the total protein in the supernatant equals the amount of protein adsorbed by different materials.

### 2.5 Cytotoxicity

High-glucose DMEM containing 10% fetal bovine serum (FBS) was used to extract different groups of materials (200 mg/mL) at 37°C for 72 h, and then the samples were centrifuged at 13,000 rpm at 4°C for 10 min to obtain extracts. MC3T3-E1 cells were implanted in 96-well cell culture plates at a density of 5 × 10^3^/mL. The material extracts were diluted to 2, 4, 6, 8, and 16 times and then added to the cells. The group without the added extract was set as the control group. After 24 h of culture, the medium was replaced with 10% CCK-8 (Beyotime, China) and incubated for 2 h. The OD_450_ value was measured using a plate reader (Tecan M200, Switzerland). The cell proliferation rate was calculated using Eq. 1. Finally, the cells were stained using a live and dead cell staining kit (Beyotime, China).

$$\text{Cell proliferation rate }\left(\%\right)=\frac{{OD}_{S}}{{OD}_{C}}\times 100\%.$$ (1)

Here, OD_s_ represents the OD_450_ value of the sample and OD_c_ represents the OD_450_ value of the control group.

### 2.6 Preparation of Gd&Si-HA/PLGA composite materials

Gd&Si-HA NPs of different weights were added to 10 mL N-methylpyrrolidone (NMP), and the ultrasonic dispersion of the NPs was uniform. A measure of 1.6 g PLGA was added to the above solution and stirred until PLGA was completely dissolved. Then, the solution was poured on the surface of the glass slide, and the glass slide coated with the Gd&Si-HA/PLGA composite was soaked in water for solvent replacement. Finally, the Gd&Si-HA/PLGA composite film was prepared for subsequent experiments. The other part of the Gd&Si-HA/PLGA solution was taken in a 2-mL syringe and withdrawn in water for solvent replacement to prepare a scaffold for the MRI experiment *in vitro*.

### 2.7 *In vitro* MRI

Gd&Si-HA/PLGA composite scaffolds with Gd molar concentrations of 0.19, 0.38, 0.75, and 1.5 mM were prepared using 1.5Gd&Si-HA and 0.8Gd&Si-HA NPs for the measurement of MRI effect. The content of 1.5Gd&Si-HA, 0.8Gd&Si-HA NPs, and PLGA in 2 cm^3^ of different Gd&Si-HA/PLGA composite scaffolds is shown in Supplementary Table S2. The material was dissolved with NMP solution and then added to the aqueous solution using a 2-mL syringe for solvent displacement to prepare the scaffold. The HA/PLGA scaffold was used as the negative control. The MRI effect of the scaffold was measured using a 1.2 T MRI scanner (HT-MRSI50-50KY, Shanghai, China).

### 2.8 Osteogenic differentiation ability

MC3T3-E1 cells were implanted on the glass slide coated with the Gd&Si-HA/PLGA composite at a density of 1 × 10^4^ cells. Half of the cells were stimulated by a static magnetic field of 80 mT, and the other half were not stimulated by the magnetic field. After 4 days of culture, the total RNA was extracted, cDNA was synthesized using a reverse transcription kit (Takara, Japan), and collagen I (COL-I), osteocalcin (OCN), runt-related transcription factor 2 (Runx2), and bone morphogenetic protein-2 (BMP-2) were detected using real-time PCR. The gene primers are shown in Supplementary Table S3. COL-I expressed by cells was stained using immunofluorescence staining. After 7 days of culture, ALP expressed by cells was stained using a kit (Beyotime, China). After 14 days of culture, the cells were stained for calcium deposits using Alizarin Red staining kits (Beyotime, China). The S and N elements were observed by EDS mapping (ZEISS, Germany) after 14 days of cell culture.

### 2.9 Statistical analysis

The results of the analysis were represented as mean ± standard deviation. The test for the significant difference was performed using Student’s t-test in OriginPro 8.5.1 (OriginLab Corp., United States). The tests were performed at a *p* < 0.05 level.

## 3 Results and discussion

### 3.1 Chemical structure and phase composition analysis

**FIGURE 1:**
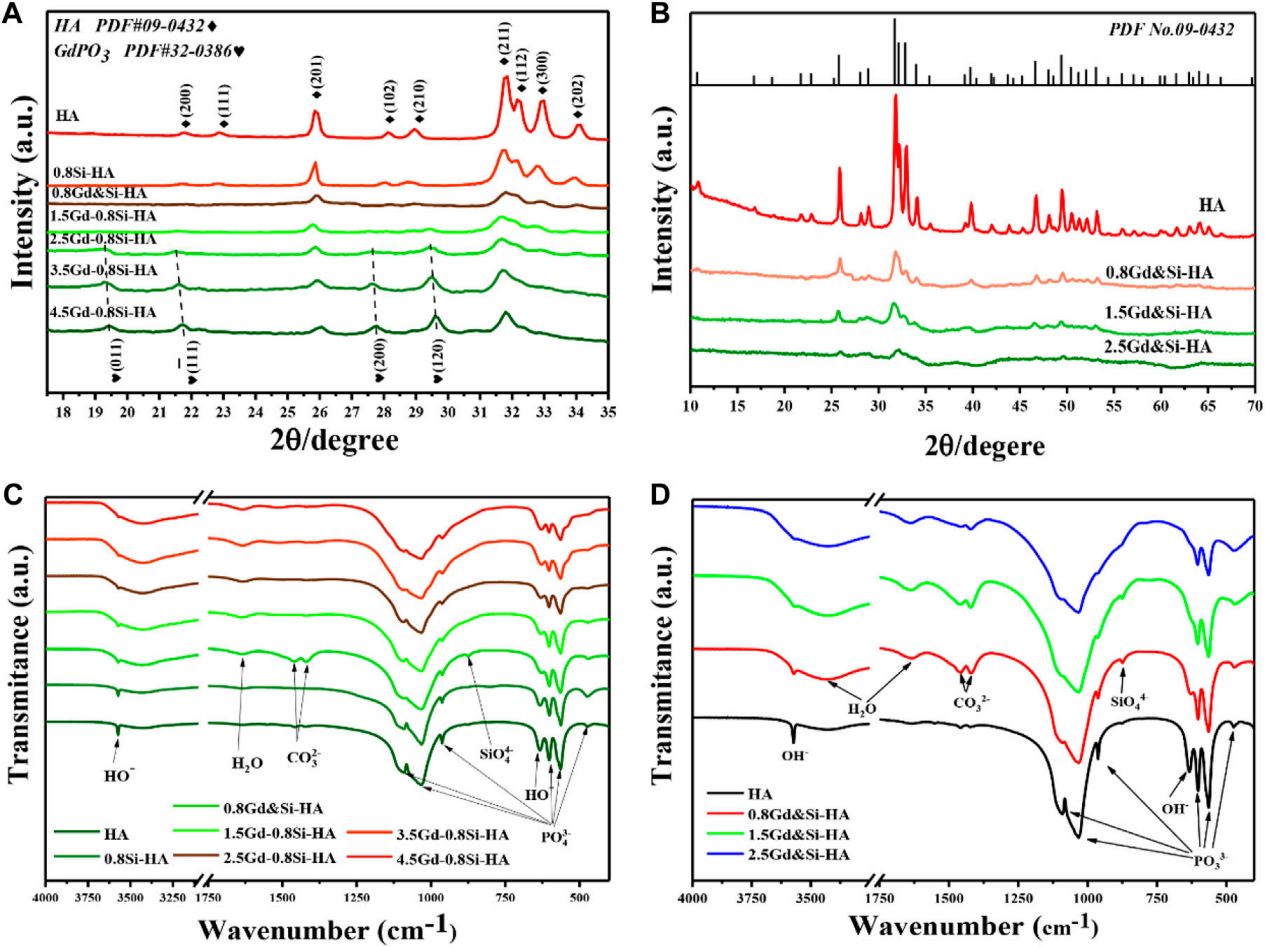
**(A,B)** XRD patterns and **(C,D)** FTIR spectra of a series of HA NPs containing different Si and Gd doping amounts.

Figures 1A, B show the XRD patterns of synthetic products of the doped HA samples with the tailored Gd and Si amount. As shown in Figure 1A, when the Si doping amount was fixed at 0.8Si-HA, the products of low Gd doping amount (0.8Gd–0.8Si-HA) could be identified as a pure HA phase (PDF No. 09-0432). However, as the Gd amount increased (1.5/2.5/3.5/4.5Gd–0.8Si-HA), the characteristic peaks at 19.4°, 21.7°, 27.6°, and 29.5° corresponding to (011), (−111), (200), and (120) of second-phase gadolinium phosphate (PDF No. 32-0386) appeared, which implicates that the Gd doping upper limit is 0.8Gd–0.8Si-HA. On the contrary, as shown in Figure 1B, all co-doping products, including high-doping samples, were identified as a pure HA phase in the absence of any diffraction peaks of secondary phase. Moreover, as the co-doping amount increased, the characteristic diffraction peak intensities decreased and the half-peak widths increased, indicating that doping leads to a significant decrease in crystallinity. Furthermore, the comparison of the XRD patterns revealed that 1.5Gd–1.5Si-HA samples with more doping elements show a cleaner HA phase composition than 1.5Gd–0.8Si-HA samples.

The FTIR spectra of the synthesized samples revealed their chemical compositions. Figure 1C shows the spectra of samples doped with different Gd contents, and Figure 1D shows the spectra of co-doping products. All samples exhibited the characteristic adsorption bands of hydroxyapatite. In brief, the bending vibrations of the O-P-O bond (1,100–960 and 560–605 cm^−1^), stretching vibrations of the P-O bond (1,029 cm^−1^ and 960 cm^−1^), the stretching vibrations of OH^−^group (635 cm^−1^), bending modes of the adsorbed water (3,427 and 1,635 cm^−1^), and stretching vibrations of the OH^−^group (3,570 cm^−1^) were in close agreement with those obtained in previous studies. In addition, low-intensity bands corresponding to the vibrational modes of the Si-O-Si bond (875 cm^−1^) was observed and regarded as specific features of silicic ion, which provides strong evidence of the successful doping of Si. Moreover, with the increase in the Gd doping amount (Figure 1C), characteristic carbonate peaks were additionally observed at 1,550–1,410 cm^−1^, which might have occurred from the dissolved carbon dioxide in phosphate solutions. In addition, the bending mode of the absorbed water located at 3,427 cm^–1^ was significantly distinct as the amount of Gd increased. On the other hand, the characteristic carbonate peaks and absorbed water in the FTIR spectra of co-doping samples were also obvious, as shown in Figure 1D. The analysis of the XRD results showed that this phenomenon could be attributed to the increase in lattice distortion caused by Gd doping, the decrease in crystallinity, and the enhanced ability of the sample to adsorb and bind water.

To further quantitatively analyze the doping efficiency of the doped products, the Gd wt% of the samples was investigated by ICP, and the results are given in Supplementary Table S1. The Gd content of the 1.5Gd–0.8Si-HA and 2.5Gd–0.8Si-HA samples was 14.4 and 27.3 _wt_%, respectively, while the Gd content of 1.5Gd-1.5Si-HA and 2.5Gd–2.5Si-HA samples was 16.5 and 34.0 _wt_%, respectively. As expected, when the Gd addition was consistent, the co-doping samples showed a much higher doping dose.

These phase and chemical composition results suggested that Gd and Si had been substituted into the HA lattice through the mechanism expressed in the following equation:

$$\begin{array}{l} \left(10-x\right)Ca^{2+}+xGd^{3+}+\left(6-x\right)PO_{4}^{3-}+xSiO_{4}^{4-}+2OH^{-}\\ \to Ca_{\left(10-x\right)}Gd_{x}{\left(PO_{4}\right)}_{\left(6-x\right)}{\left(SiO_{4}\right)}_{x}{\left(OH\right)}_{2} \end{array}$$

Furthermore, the physical and chemical performance of the product provided proof of our concept that the equimolar ratio co-doping method can indeed reduce lattice distortion caused by charge nonconservation.

### 3.2 Microstructure analysis

**FIGURE 2:**
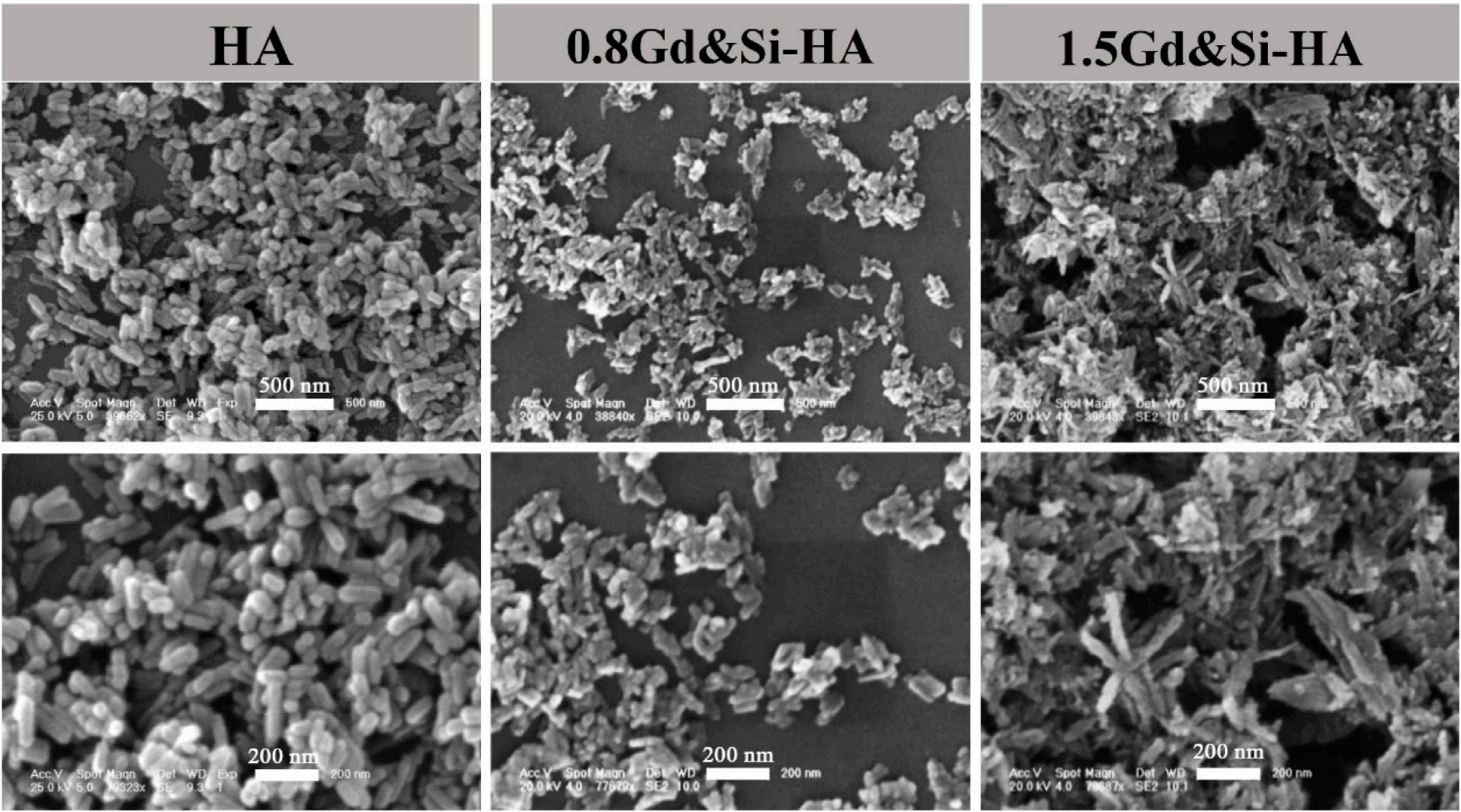
Micromorphology of HA, 0.8Gd&Si-HA, and 1.5Gd&Si-HA NPs.

SEM images (Figure 2) show the microscopic morphology of Gd&Si-HA NPs with different Si and Gd contents. It is evident from the SEM images that HA shows a short, rod-like morphology with 30 nm diameter and less than 100 nm length, and after Gd and Si co-doping (0.8Gd–0.8Si-HA), the grains appear smaller and the agglomeration phenomenon is obvious. Furthermore, the co-doping sample with a high doping dose (1.5Gd&Si-HA) showed a cluster-like morphology with further aggravated agglomeration. The above morphological change in the co-doped samples was speculated to be associated with high surface energy activated by doping.

### 3.3 Magnetic and surface potential characterization

**FIGURE 3:**
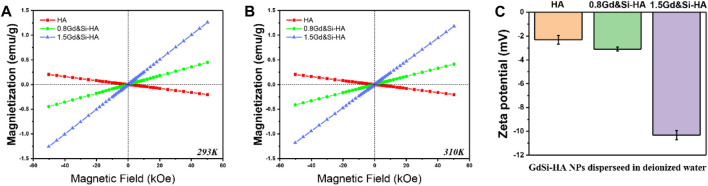
**(A,B)** Magnetic characterization of HA and Gd&Si-HA NPs at room temperature and body temperature, respectively, and **(C)** surface potential of HA and Gd&Si-HA NPs (*n* = 3).

As shown in Figures 3A, B, the magnetic properties of HA and Gd&Si-HA NP materials were measured at room temperature and body temperature. The magnetization curve of HA samples was a straight line distributed in II and IV quadrants, which indicates that the HA NPs are characterized as a type of diamagnetic material. On the contrary, the magnetization curves of HA samples after Gd&Si co-doping were distributed in the I and III quadrants in a straight line and passed through the origin, indicating that Gd&Si-HA NPs are a type of paramagnetic material. Moreover, the magnetization curve shifted to the Y-axis with the increase in the Gd&Si co-doping dose, indicating that the magnetic properties of the material were enhanced with the increase in the Gd doping dose. The trends of magnetic properties exhibited by the product at body temperature (Figure 3B) were consistent with those at room temperature (Figure 3A).

Figure 3C shows the surface potentials of HA and Gd&Si-HA NPs. HA has a negative surface potential, and Gd&Si-HA also has a negative surface potential after Gd&Si co-doping. The surface potential of the co-doped sample 0.8Gd&Si-HA NPs is similar to that of HA NPs, while the surface potential of 1.5Gd&Si-HA NPs is more negative. It is speculated that this may be related to the crystal distortion caused by doping and the decrease in the crystal size of the material.

### 3.4 Protein adsorption capacity

**FIGURE 4:**
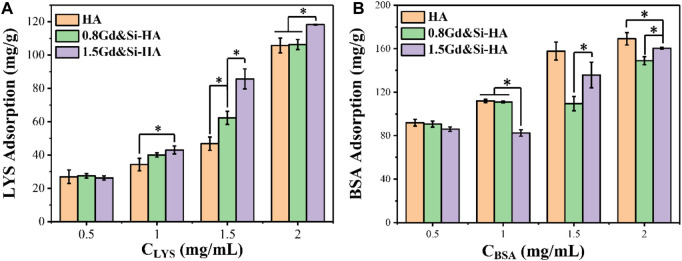
Adsorption capacity of HA, 0.8Gd&Si-HA, and 1.5Gd&Si-HA NPs to different concentrations of **(A)** LYS and **(B)** BSA (*n* = 3, *p < 0.05).

To further reveal the protein affinity of the as-prepared products, the proteins with different isoelectric points (PIs) as models (LYS PI 10.7 and BSA PI 4.8) were employed to evaluate the adsorption capacity. As shown in Figure 4, the adsorption capacity of HA, 0.8Gd&Si-HA, and 1.5Gd&Si-HA NPs with LYS and BSA was determined. On the one hand, when the concentration of LYS is higher than 1 mg/mL, the adsorption capacity of LYS in the co-doped HA sample is significantly higher than that of HA, and the adsorption capacity of LYS increases with the increase in the doping amount. Since LYS was positively charged in pH 7.4 PBS, 1.5Gd&Si-HA NPs possessed higher affinity than 0.8Gd&Si-HA and HA NPs, which is attributed to the more negative surface potential of 1.5Gd&Si-HA NPs (Figure 3C). On the other hand, as a result of surface potential, when Gd&Si-HA NPs were incubated with BSA solutions of different concentrations, the adsorbed BSA of co-doped HA NPs was less than that of HA. However, when the concentrations of BSA were higher than 1.5 mg/mL, the adsorption of BSA on the surface of the 1.5Gd&Si-HA NP sample group was higher than that on the 0.8Gd&Si-HA NP sample group. The principal reason for this phenomenon was smaller particle size and higher active surface energy of 1.5Gd&Si-HA than 0.8Gd&Si-HA.

Gd^3+^ ions doped with HA can improve its physicochemical properties and contribute to the new biological functions of the materials. Yuan et al. (2021) synthesized La-doped HA (La-HA) and Gd-doped HA (Gd-HA) and evaluated the effects of different ion doping concentrations on the physicochemical and biological properties of HA. The crystal structure, particle size, and zeta potential phase of Gd-HA are similar to those of La-HA, but Gd-HA can adsorb more serum proteins in the medium and inhibit the formation of new apatite layers on its surface. Liu et al. (2019) developed a GD-doped HA nanorod drug delivery system, and the results showed that this type of material has high loading capacity and pH response to doxorubicin (DOX).

### 3.5 Cytocompatibility

**FIGURE 5:**
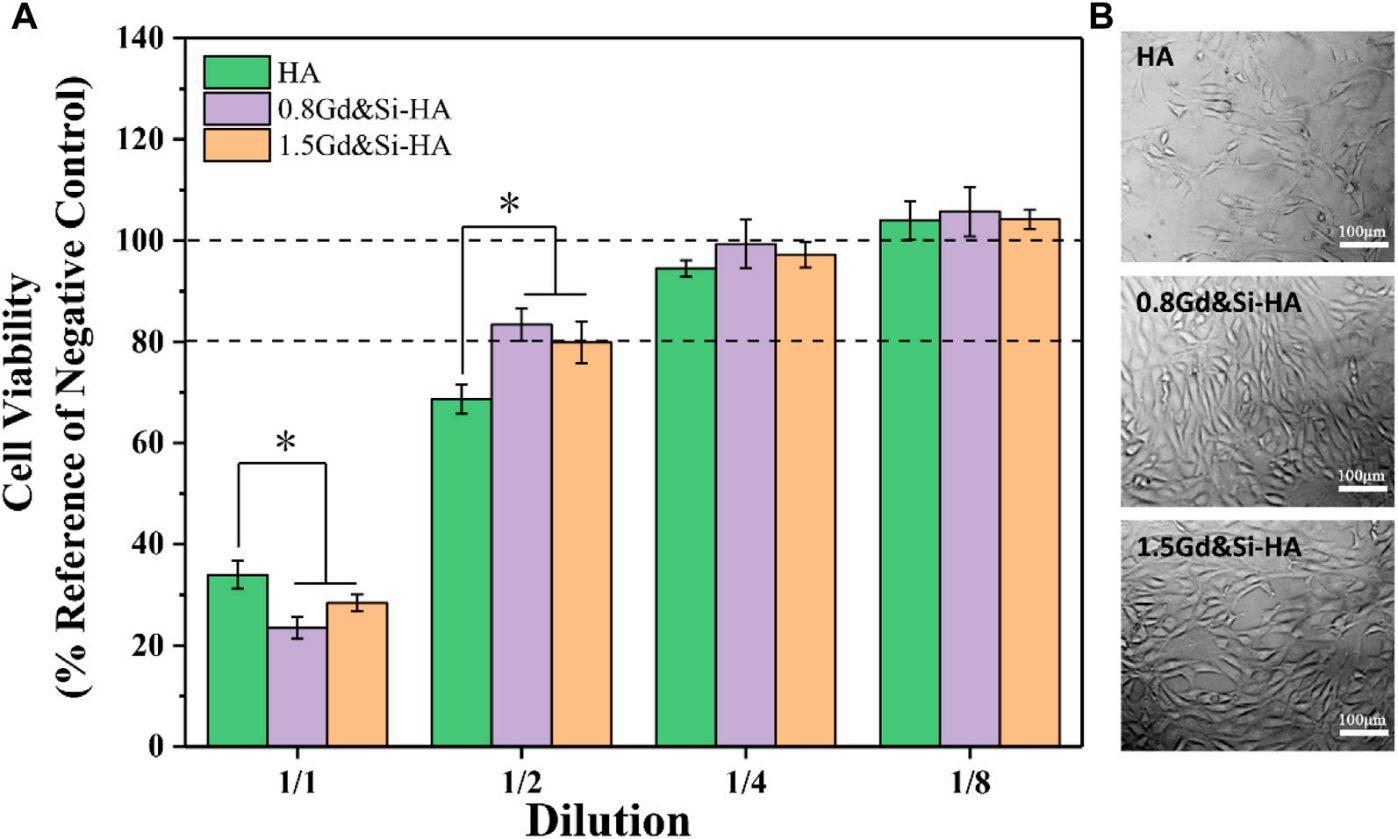
Cytocompatibility of HA, 0.8Gd&Si-HA, and 1.5Gd&Si-HA NP extracts at different dilution ratios. **(A)** CCK-8 assay (*n* = 3, *p < 0.05); **(B)** cell morphology images at 1/2 dilution.

As shown in Figure 5A, all samples showed obvious toxicity when the cells were cultured in the stock extract solution. The cell survival rate of HA, 0.8Gd&Si-HA, and 1.5Gd&Si-HA NP was 33.95%, 23.48%, and 28.42%, respectively. When the cells were cultured in 1/2 dilution for 24 h, the cell survival rate of the HA group was lower than 80%, but the cell survival rate of the co-doped sample was higher than 80%, indicating that the Ca, Gd, P, and Si elements released by the decomposition of the co-doped sample played a significant role in promoting cell proliferation. When cultured in 1/4 dilution for 24 h, the cell survival rate of all sample groups was higher than 90%, and 0.8Gd&Si-HA NPs had the highest cell survival rate of 99.32%. However, the cell survival rate of the 1.5Gd&Si-HA group was 97.20%, which was higher than that of the HA groups. When cultured in 1/8 dilution for 24 h, the cell survival rates of all groups were higher than 100%. As shown in Figure 5B, MC3T3-E1 cell morphology was captured after 24 h. In the HA group, the number of cells was small. In the 0.8Gd&Si-HA group, the number of cells was the highest and the cell morphology was healthy and spindle-shaped. In the 1.5Gd&Si-HA group, the number of cells was slightly lower than that in the 0.8Gd&Si-HA group.

### 3.6 MRI effect *in vitro*

**FIGURE 6:**
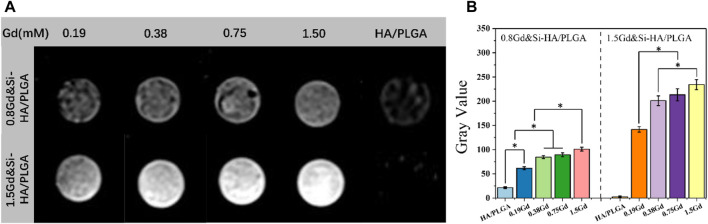
**(A)** MRI of the Gd&Si-HA/PLGA composite scaffold doped with different Gd molar concentrations; **(B)** MRI gray value of different composite scaffold (*n* = 3, *p < 0.05).

To investigate the MRI signal enhancement effects of the as-obtained nano-composite scaffolds, the MRI effect of various Gd concentrations (range 0.19–1.5 mM) was obtained in PBS on a 1.2 T MRI scanner, where the element content of Gd of the scaffold samples was detected by ICP-MS analysis. As shown in Figure 6, the HA/PLGA scaffold in MRI T_1_ was black–gray without imaging effect. The Gd&Si-HA/PLGA group showed the effect of MRI T_1_ phase imaging. Under the same Gd molar concentration, the 1.5Gd&Si-HA/PLGA sample group looks brighter than the 0.8Gd&Si-HA/PLGA sample group, resulting in a more obvious MRI effect. Moreover, the imaging effect of the 1.5Gd&Si-HA/PLGA group showed a dose-dependent relationship with the Gd molar concentration. MRI is a powerful technique for obtaining tomographic images of biological targets in a noninvasive manner with a high spatial resolution. In MRI, contrast agents are employed to enhance the visualization of the differences between normal and diseased tissues. The image contrast in MRI is related to the relaxation process of hydrogen nuclei of water molecules. It is governed by three parameters: proton density, longitudinal relaxation time T_1_, and transverse relaxation time T_2_. Gd-based T_1_ contrast agents, such as gadopentetic acid (Gd-DTPA), could shorten the longitudinal relaxation time of protons within water molecules under the influence of rapid relaxation of unpaired electrons within the agents, resulting in the signal enhancement of T_1_-weighted MR images in the application form of intravenous injection. Previous studies have shown that under the same Gd concentration, Gd-HA and Gd-DTPA have similar T_1_ MRI effects, and this effect becomes stronger with the increase in the Gd concentration (Liu et al., 2019). However, for the application of contrast agents in polyester-based composite materials, specifically PLGA-, PLA-, and PCL-based composites, the hydrophobic properties of the matrix significantly reduced the proton density, resulting in a low MRI contrast effect. In this study, the co-doped samples possessed an enhanced T_1_ contrast enhancement effect in a Gd dose-dependent manner, suggesting that the co-doped samples with a high Gd dose might have potential applications as MRI contrast agents. Moreover, the 1.5Gd&Si-HA/PLGA group had the strongest T_1_ contrast enhancement effect; at the same time, Gd had the concentration comparable to that of the 0.8Gd&Si-HA/PLGA and HA/PLGA groups (Figure 6). As shown in Figure 1, after Gd and Si doping, the distortion in the lattice was accelerated, and as a result, the particle size became smaller and the surface potential became more negative, enhancing the binding of water molecules, which could significantly increase the proton density in hydrophobic polyester and amplify the T_1_ contrast enhancement effect. Therefore, 1.5Gd&Si-HA NPs are an excellent T_1_ MRI contrast agent for *in vivo* imaging of bone repair scaffolds. In addition, Gd can also be co-doped with other rare earth elements to contribute to HA new functions. Chen et al. (2012) developed a type of Eu^3+^/Gd^3+^ dual-doped calcium–phosphorus materials, which exhibit vesicle-like nanospheres and strong near-infrared (NIR) fluorescence imaging characteristics.

### 3.7 Osteogenic differentiation

**FIGURE 7:**
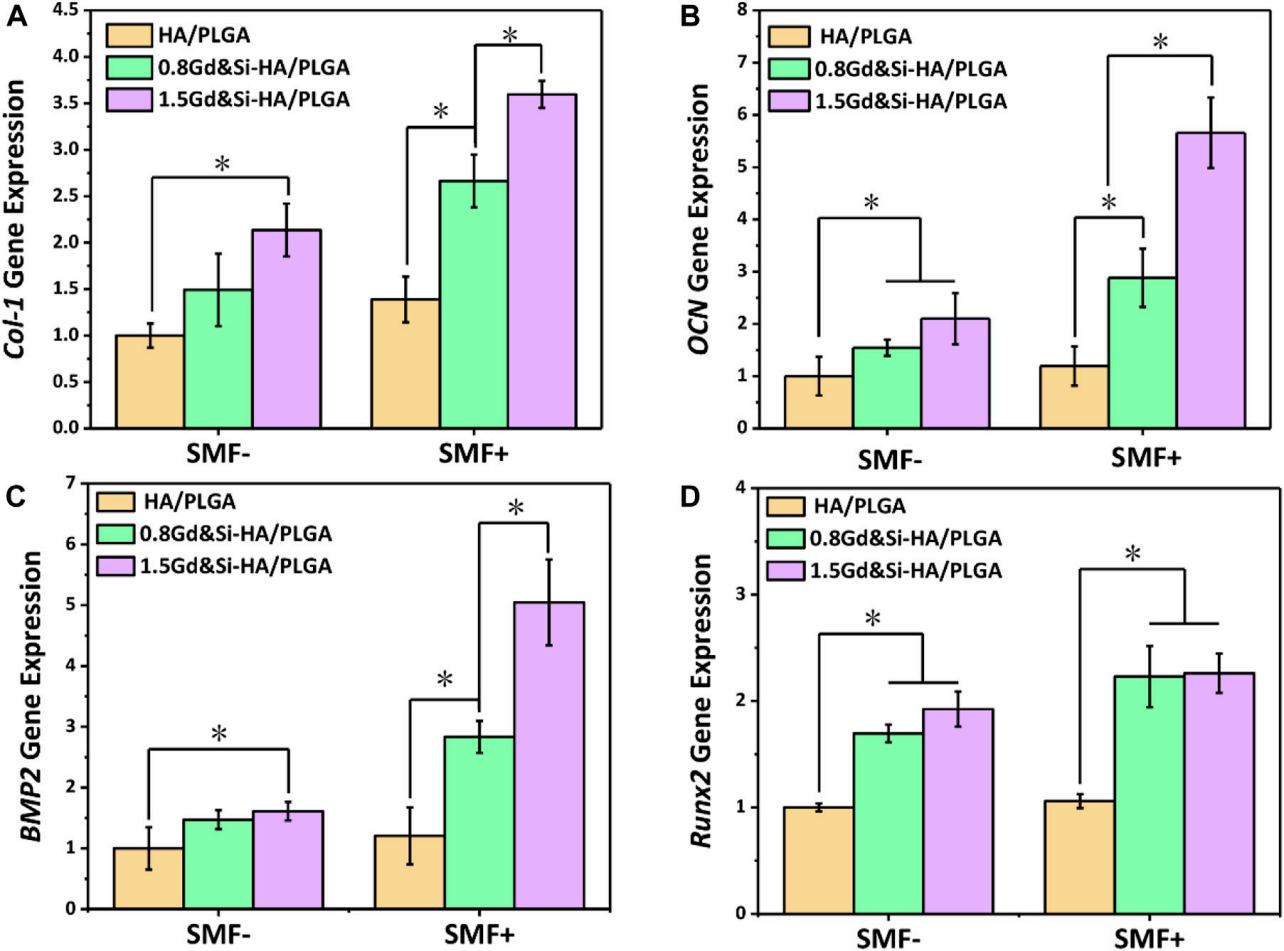
Expression levels of osteogenic-related genes in MC3T3-E1 cells under SMF+ and SMF− stimulation conditions at 4 days. **(A)** COL-I, **(B)** OCN, **(C)** BMP-2, and **(D)** Runx2 (*n* = 3, *p < 0.05).

As shown in Figure 7A, the expression level of COL-1 was the lowest in the HA/PLGA group, and the expression level of COL-1 in the group containing Gd&Si-HA NPs was significantly increased compared with that in the HA/PLGA group. The COL-I expression levels in the 1.5Gd&Si-HA/PLGA group were 1.35 and 2.59 times higher than those in the 0.8Gd&Si-HA/PLGA and HA/PLGA groups under SMF+ stimulation, respectively. As shown in Figure 7B, the trend of the OCN expression level was basically consistent with COL-I. Under SMF+ stimulation, the OCN expression level in the 0.8Gd&Si-HA/PLGA group and 1.5Gd&Si-HA/PLGA group was 1.87 and 2.69 times higher than that under the SMF− condition, respectively. Under SMF+ stimulation conditions, the expression level of BMP-2 was consistent with that of COL-1 and OCN, and Gd&Si-HA NPs contained in the scaffold could significantly improve the expression level of BMP-2, which showed a concentration-dependent relationship with Gd concentration (Figure 7C). Under SMF+ stimulation conditions, Runx2 expression levels in the Gd&Si-HA/PLGA scaffold increased. However, the expression levels of Runx2 in the 0.8Gd&Si-HA/PLGA group and 1.5Gd&Si-HA/PLGA group were not significantly different (Figure 7D).

**FIGURE 8:**
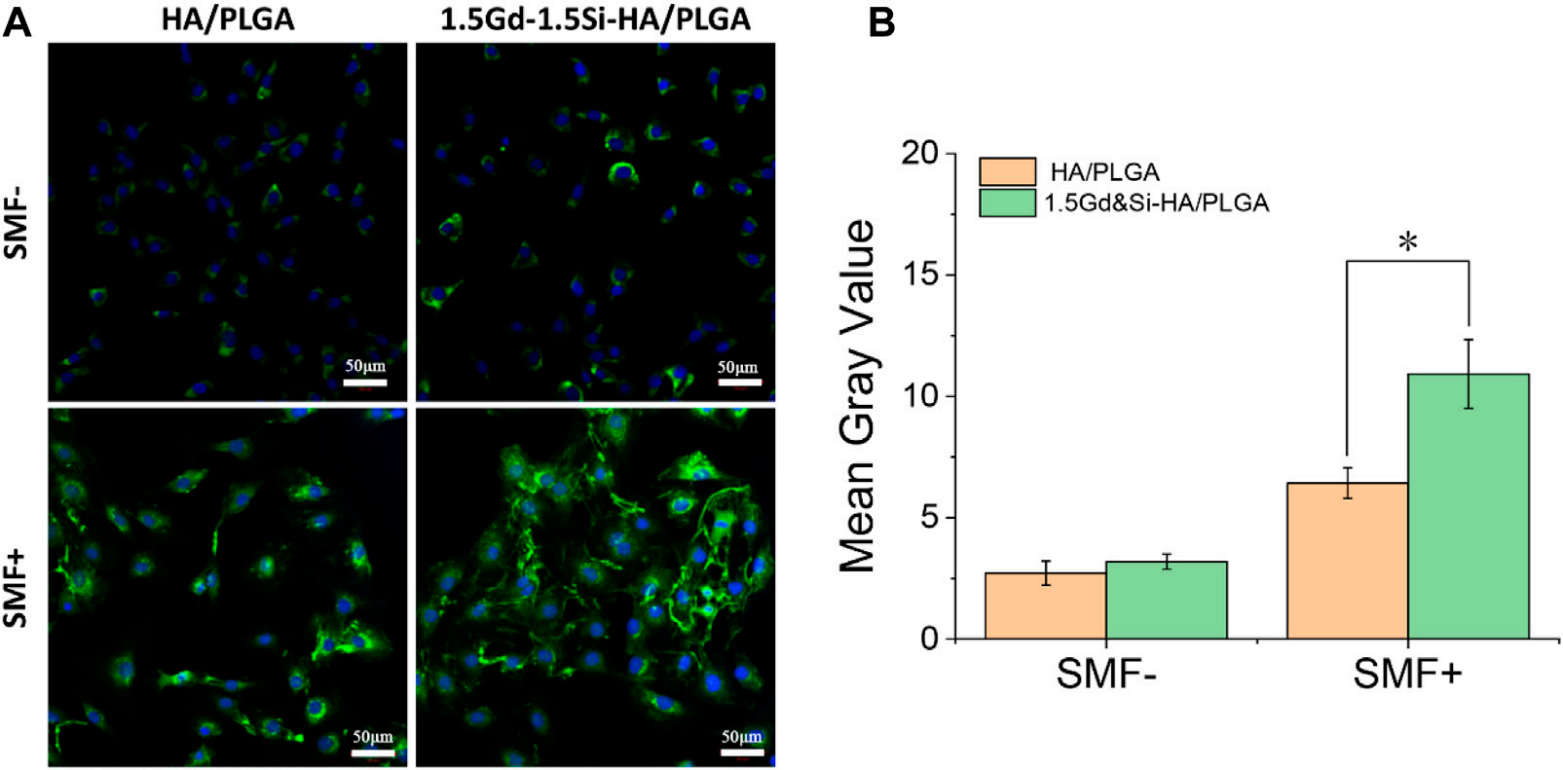
**(A)** COL-I immunofluorescence image and **(B)** quantitative analysis of MC3T3-E1 cells cultured on HA/PLGA and 1.5Gd&Si-HA/PLGA surfaces for 4 days under SMF+ and SMF− conditions (*n* = 3, *p < 0.05).

As shown in Figure 8A, immunofluorescence images showed the expression of COL-I protein in MC3T3-E1 cells growing on the surface of HA/PLGA and 1.5Gd&Si-HA/PLGA under SMF+/SMF− stimulation conditions. The results of immunofluorescence image analysis were basically consistent with those of *q*PCR. Under the SMF− condition, COL-1 expression in the 1.5Gd&Si-HA/PLGA group was slightly higher than that in the HA/PLGA group. However, the COL-I expression level was significantly higher than that in the HA/PLGA group under the SMF+ stimulation condition. As shown in Figure 8B, the quantitative analysis of COL-I showed that the mean gray value of the 1.5Gd&Si-HA/PLGA group was 1.7 times higher than that of the HA/PLGA group under the SMF− condition.

**FIGURE 9:**
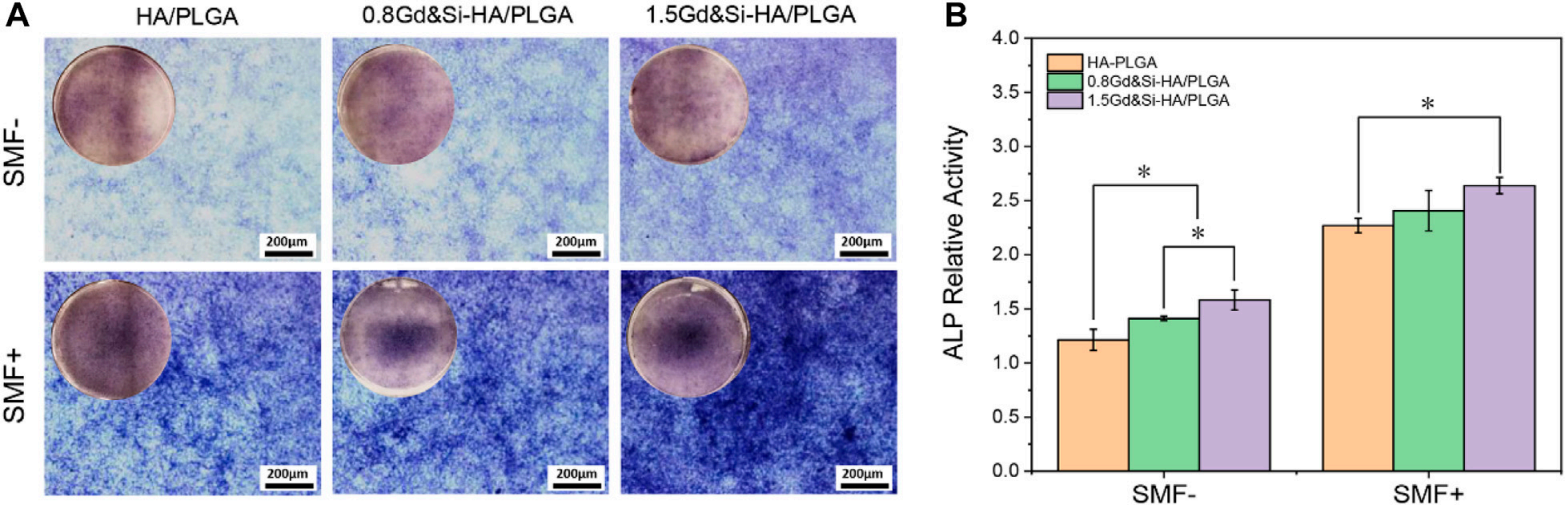
**(A)** ALP staining and **(B)** quantitative analysis of MC3T3-E1 cells cultured on HA/PLGA, 0.8Gd&Si-HA/PLGA, and 1.5Gd&Si-HA/PLGA surfaces under SMF+/SMF− conditions for 7 days (*n* = 3, *p < 0.05).

The results of ALP staining showed that the expression of ALP in all groups of cells under SMF+ stimulation conditions was significantly higher than that under SMF− simulation conditions (Figure 9A). Under SMF−/SMF+ conditions, the expression of ALP in the Gd&Si-HA NP group was significantly higher than that in the HA/PLGA group. The expression level of ALP in the 1.5Gd&Si-HA/PLGA group was higher than that in the 0.8Gd&Si-HA/PLGA group. As shown in Figure 9B, the quantitative results of ALP in each group were basically the same as those for ALP staining. Under SMF+ conditions, the expression levels of ALP in the 1.5Gd&Si-HA/PLGA and 0.8Gd&Si-HA/PLGA groups were 1.66 and 1.70 times higher than those under SMF− conditions, respectively.

**FIGURE 10:**
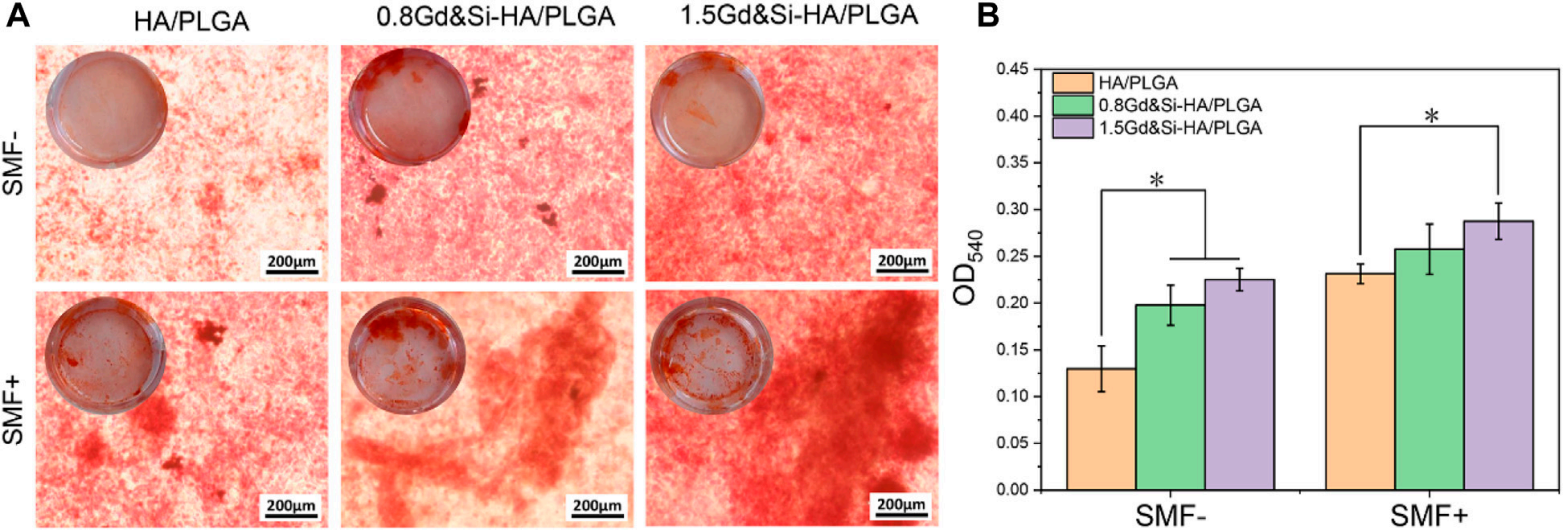
**(A)** Alizarin Red staining and **(B)** calcium ion quantitative analysis of MC3T3-E1 cells cultured on HA/PLGA, 0.8Gd&Si-HA/PLGA, and 1.5Gd&Si-HA/PLGA surfaces under SMF+/SMF− conditions for 14 days (*n* = 3, *p < 0.05).

As shown in Figure 10A, calcium deposition of cells on the surface of different scaffolds was detected under SMF+/SMF− conditions at 14 days. The results of Alizarin Red staining showed that all cells of the groups could promote calcium deposition under the SMF+ condition. Interestingly, scaffolds containing Gd&Si-HA NPs showed more significant effects on cellular calcium deposition under SMF+ stimulation conditions. The effect of calcium deposition in the 1.5Gd&Si-HA/PLGA group was significantly better than that in the 0.8Gd&Si-HA/PLGA group. Calcium quantitative results showed that under SMF+ conditions, the effects of calcium deposition in the 0.8Gd&Si-HA/PLGA and 1.5Gd&Si-HA/PLGA groups were 1.11 times and 1.24 times higher than those in the HA/PLGA group, respectively (Figure 10B).

Although HA has good osteoconductive property, it has poor osteoinductive property (Arinzeh et al., 2005). In order to improve the osteoinductive ability of the HA material, Si was often doped into HA. Although Si is a trace element in the human body, it shows strong ability to promote cell osteoblastic differentiation and promote angiogenesis *in vivo* (Guth et al., 2006; Casarrubios et al., 2020; Fu et al., 2020). Fu et al. (2020) prepared Si-doped HA coatings on the surface of Ti using electrochemical deposition (ED). This type of Si-HA layer exhibited stronger apatite deposition and higher BSA adsorption capacity. The results of an *in vitro* cell experiment showed that the Si-HA layer could promote the expression of osteogenic-related genes in MC3T3-E1 cells and angiogenesis-related gene expression in HUVECs (Fu et al., 2020). In this study, Si and Gd were co-doped into HA, where Si mainly played a role in promoting bone formation and Gd mainly played a role as an MRI contrast agent *in vivo*. The results showed that the doping of Si significantly promoted the upregulated expression of osteogenic genes (Figure 7). After Gd and Si doping, the distortion in the lattice was accelerated, and as a result, the particle size became smaller and the surface potential became more negative, enhancing the binding of water molecules, which could significantly increase the proton density in hydrophobic polyester and amplify the T_1_ contrast enhancement effect.

## 4 Conclusion

In order to improve the osteoinductive ability and MRI ability of HA, Si and Gd were co-doped into HA. The XRD pattern results show that co-doping with an equal molar ratio can avoid crystal defects and increase the doping dose of Gd. No significant cytotoxicity was found in both 0.8Gd&Si-HA/PLGA and 1.5Gd&Si-HA/PLGA groups. The 1.5Gd&Si-HA/PLGA composite materials showed a good MRI effect in the Gd concentration range of 0.19–1.5 mM. The 1.5Gd&Si-HA/PLGA composite materials also showed excellent osteoinductive ability. Under SMF+ conditions, the 1.5Gd&Si-HA/PLGA composite materials can obviously promote the expression of osteogenic-related genes, the level of ALP, and cell mineralization compared to SMF− conditions. The bone repair scaffold prepared using 1.5Gd&Si-HA/PLGA composite materials has a broad application prospect in the field of bone tissue engineering.

## Data Availability

The original contributions presented in the study are included in the article/Supplementary Material; further inquiries can be directed to the corresponding authors.
