# Supplementary material for: Silicon and gadolinium co-doped hydroxyapatite/PLGA scaffolds with osteoinductive and MRI dual functions
Source: Front Bioeng Biotechnol. 2024 Jan 9;11:1310017. doi: 10.3389/fbioe.2023.1310017 (PMC10807042; doi:10.3389/fbioe.2023.1310017)
Supplement: Supplementary file 1 [file Table1.DOCX]

**Supplementary material**

**Silicon and gadolinium co-dopped hydroxyapatite/PLGA scaffolds with osteoinductive and MRI imaging dual functions**

Shaodong Xie^1^, Min Guo^2^, Deming Zeng^1^, Hanwen Luo^1^, Ping Zhong^1^, Zixuan Deng^3^, Yu Wang^2,*^, Zhiqiang Xu^1,*^, Peibiao Zhang^2^

*^1^ Department of Rehabilitation Medicine, Foshan Hospital of Traditional Chinese Medicine, Foshan, 528000, PR China*

*^2^ Key Laboratory of Polymer Ecomaterials, Changchun Institute of Applied Chemistry, Chinese Academy of Sciences, Changchun, 130022, PR China*

*^3^ Graduate student of the Eighth Clinical Medical College of Guangzhou University of Chinese Medicine, Guangzhou, 528051, PR China*

*Correspondence should be addressed to Zhiqiang Xu (xuzq@fshtcm.com.cn) and Yu Wang (wydna@ciac.ac.cn).

Table S1. the proportion of reagents in different groups of materials in hydrothermal reaction.

The concentration of Ca(NO_3_)_2_·4H_2_O, Gd(NO_3_)_3_·6H_2_O and (NH_4_)_2_HPO_4_ were 1M, 0.5M and 0.6 M, respectively.

| **Samples** | **Ca/mL**  **Ca(NO_3_)_2_·4H_2_O** | **Gd/mL**  **Gd(NO_3_)_3_·6H_2_O** | **P/mL**  **(NH_4_)_2_HPO_4_** | **Si/uL**  **Si(OCH_2_CH_3_)** | **(Ca+Gd)/(P+Si)**  **Theoretical** | **Gd /_wt_%**  **Calculated by ICP** |
| --- | --- | --- | --- | --- | --- | --- |
| HA | 2 | 0 | 2 | 0 | (2)/(1.2) | -- |
| 0.8Si-HA | 2 | 0 | 1.73 | 35.72 | (2)/(1.04+0.16) | -- |
| 1.5Si-HA | 2 | 0 | 1.5 | 66.98 | (2)/(0.9+0.3) | -- |
| 0.8Gd&Si-HA | 1.84 | 0.32 | 1.73 | 35.72 | (1.84Ca+0.16Gd)/(1.04+0.16) | 9.2 |
| 1.5Gd-0.8Si-HA | 1.7 | 0.6 | 1.73 | 35.67 | (1.7+0.3)/(1.04+0.16) | 14.4 |
| 2.5Gd-0.8Si-HA | 1.5 | 1 | 1.73 | 35.67 | (1.5+0.5)/(1.04+0.16) | 27.3 |
| 3.5Gd-0.8Si-HA | 1.3 | 1.4 | 1.73 | 35.67 | (1.3+0.7)/(1.04+0.16) | 40.6 |
| 4.5Gd-0.8Si-HA | 1.1 | 1.8 | 1.73 | 35.67 | (1.1+0.9)/(1.04+0.16) | 38.4 |
| 1.5Gd&Si-HA | 1.7 | 0.6 | 1.5 | 66.98 | (1.7+0.3)/(0.9+0.3) | 16.5 |
| 2.5Gd&Si-HA | 1.5 | 1 | 1.17 | 111.65 | (1.5+0.5)/(0.7+0.5) | 34.0 |

Table S2. The weight of 0.8Gd&Si-HA and 1.5Gd&Si-HA NPs in 2 cm^3^ different Gd molar concentration composite scaffold.

| **Gd(mM)** | **0.19** | **0.38** | **0.75** | **1.50** |
| --- | --- | --- | --- | --- |
| 0.8Gd&Si-HA (mg) | 0.64 | 1.28 | 2.56 | 5.13 |
| 1.5Gd&Si-HA (mg) | 0.36 | 0.71 | 1.43 | 2.86 |
| PLGA (mg) | 400 | 400 | 400 | 400 |

Table S3. Sequence of osteogenic-related genes primers

| **Gene Annotation** | **Primer Sequence (5’-3’)** | **Length (bp)** | **Reference** |
| --- | --- | --- | --- |
| **COL I** | F: CGCTGGCAAGAATGGCGATC | 20 | NM_007742.3 |
|  | R: ATGCCTCTGTCACCTTGTTCG | 21 |  |
| **BMP-2** | F: CGCAATCTCCATGTTGTACCTAG | 23 | NM_007553.2 |
|  | R: GCTTAGGGCATGAGCTTTGAC | 21 |  |
| **OCN** | F: TGTGGAGTGTGACAATGGTG | 20 | NM_001032298.3 |
|  | R: CGCTACCTAACGGCATGACAG | 21 |  |
| **Runx2** | F: GCCCTCATCCTTCACTCCAAG | 21 | [NM_001145920.1](http://www.ncbi.nlm.nih.gov/nuccore/NM_001145920.1) |
|  | R: GGTCAGTCAGTGCCTTTCCTC | 21 |  |
| **GAPDH** | F: AATGTGTCCGTCGTGGATCTG | 21 | NM_008084.2 |
|  | R: CAACCTGGTCCTCAGTGTAGC  P: CGTGCCGCCTGGAGAAACCTGCC | 21  23 |  |
